# Current clinical applications of cardiovascular magnetic resonance imaging

**DOI:** 10.5830/CVJA-2014-021

**Published:** 2014

**Authors:** Leonie Scholtz, Z Lockhat, Andrew Sarkin

**Affiliations:** Department of Radiology, Steve Biko Hospital, Pretoria, South Africa; Department of Radiology, Steve Biko Hospital, Pretoria, South Africa; Department of Cardiology, Steve Biko Hospital, Pretoria, South Africa

**Keywords:** cardiovascular, cardiomyopathy, imaging, Africa, non-invasive

## Abstract

Cardiovascular magnetic resonance (CMR) imaging is unsurpassed in the evaluation of myocardial anatomy, function and mass. Myocardial perfusion pre- and post-stress, as well as late enhancement is increasingly used in the work-up for ischaemic heart disease, especially in establishing the presence of myocardial viability. Late enhancement patterns can contribute substantially to the diagnosis of myocarditis and various cardiomyopathies as well as infiltrative diseases and tumours. With their high incidence of cardiovascular disease, patients on the African continent could potentially benefit enormously from the proper utilisation of this exciting, continually evolving and versatile technique, via thorough didactic and clinical training as well as interdisciplinary co-operation.

## Abstract

The quality of CMR imaging has consequently improved dramatically since the first MR images of the human heart were described more than 20 years ago, and it is still advancing apace. CMR now represents one of the most versatile, non-invasive imaging modalities available, offering high spatial resolution and image contrast along with tissue characterisation and haemodynamic assessment, without applying ionising radiation, and with complete multiplanar coverage of the heart.

Good communication between the referring physician and the CMR specialist is paramount to streamline the type and order of sequences required for each particular scenario. Although new sequences are constantly being developed, the basic principles of CMR remain unaltered, as follows.

## Basic principles of CMR

## Scout imaging

Each examination starts with a series of scout views performed on each patient to establish the short- and long-axis views of the heart. These act as localisers in planning the rest of the study. The pulse sequences used for scouting are based on steady-state free-precession (SSFP). Typically, 27 scout images are acquired to define the thoracic contents, including nine parallel images in each of the axial, coronal and sagittal planes.

## Anatomical and morphological imaging

To assess anatomy and morphology, static images are required. Black-blood imaging is usually preferred because it allows clear distinguishing of the inner portion of the vessel or myocardium from blood. Half-Fourier single-shot fast-spin echo (HASTE) is a special variant of the fast-spin echo sequences, and ideal for delineation of anatomy. Anatomical and morphological information is particularly important in the diagnosis of congenital abnormalities and cardiac tumours.

## Functional imaging

Dynamic ciné CMR white blood imaging is used for global and regional left ventricular (LV) and right ventricular (RV) wall motion assessment as well as ventricular volume, ejection fraction and mass measurements. It is now widely regarded as the gold standard.^1^ The SSFP sequence is fast and ideal for white blood ciné imaging owing to its high signal-to-noise ratio and excellent ability to visualise the endocardial border. Functional analysis is especially important in the work-up of ischaemic heart disease as well as the non-ischaemic cardiomyopathies.

## Myocardial perfusion imaging

During perfusion scanning, a movie of the wash-in of gadolinium-based contrast through the myocardium is obtained (so called ‘first-pass perfusion’). The gradient echo (GRE) pulse sequence is most commonly used nowadays to visualise perfusion of the myocardium at rest or during adenosine stress testing. Perfusion defects appear as dark regions surrounded by bright contrastenhanced, normally perfused myocardium. CMR perfusion is playing an increasingly important diagnostic role in ischaemic heart disease.

## Oedema imaging

Myocardial oedema is associated with prolonged magnetic resonance relaxation time on T2-weighted pulse sequences. Static dark blood images of the myocardium can be obtained, confirming the presence or absence of oedema, which manifests as bright areas among the normal darker myocardium.

## Late gadolinium enhancement (LGE) CMR imaging

LGE images are acquired with an inversion recovery-prepared GRE or SSFP imaging pulse sequences, with images acquired 10–15 minutes following gadolinium (Gd) chelate contrast administration. Gd circulates in the extracellular space and is excluded by intact myocardial cell membranes. They accumulate in areas of abnormal myocardium, resulting in T1 shortening manifesting as higher signal intensity on T1-weighted images. Gd migrates through damaged myocitic membranes into the cells (for example, in the case of myocardial infarction) or accumulates in the enlarged interstitial space (in the case of scar tissue).

The goal of LGE imaging is to create images with high contrast between the hyper-enhanced, damaged, fibrotic or non-viable tissue and the normal myocardium. LGE patterns play an important role in viability assessment during acute or chronic myocardial infarction as well as in the setting of non-ischaemic cardiomyopathies and cardiac tumours.

## Flow/velocity imaging

Velocity-encoded (VENC) CMR imaging of blood flow is usually performed to measure velocity in the arteries, veins and across valves or shunts. With VENC CMR, a ciné series of greyscale images reflecting flow during the cardiac cycle is acquired. The grey level is proportional to the velocity of blood into or out of the measured plane. VENC CMR allows quantification of valvular stenosis or regurgitation and is used in the assessment of valvular pathology.

## Role of CMR in cardiovascular pathology

CMR plays an increasingly important role in cardiovascular pathology, as follows.

## Ischaemic heart disease

## Myocardial infarction and T2-weighted imaging

**Fig. 1.**
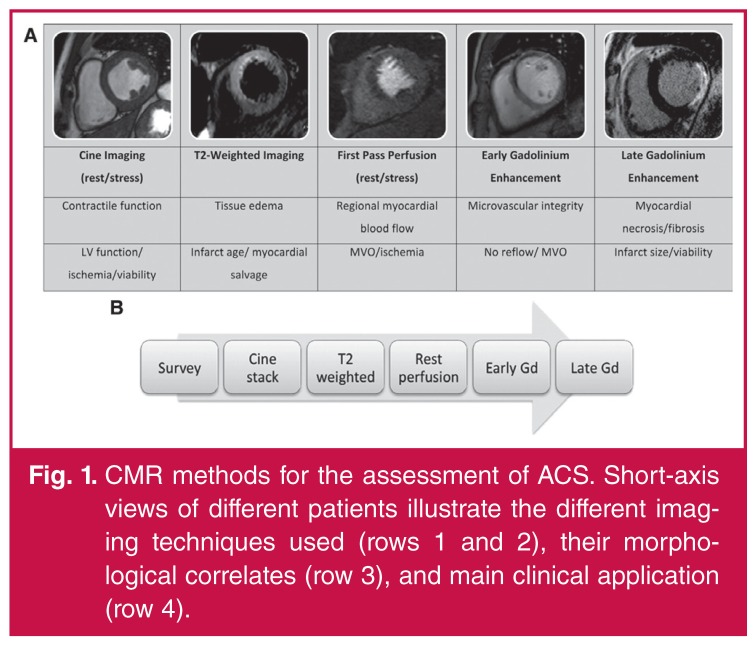
CMR methods for the assessment of ACS. Short-axis views of different patients illustrate the different imaging techniques used (rows 1 and 2), their morphological correlates (row 3), and main clinical application (row 4).

In the event of an acute myocardial infarct, myocardial oedema can be seen on T2 sequences as early as 30 minutes after the onset of ischaemia.^2^ T2-weighted CMR imaging can help to differentiate between acute and chronic myocardial infarction.^3^ CMR is consequently also useful in patients with acute chest pain of unclear aetiology with suspected acute coronary syndrome (Fig. 1).^4^,^5^ More importantly, high signal intensity on T2-weighted CMR, in the absence of LGE in the same area, reflects reversible ischaemic injury.^2^

There is excellent correlation between the area at risk (AAR) measured by T2-weighted imaging and the angiographic APPROACH score, which is an anatomically and prognostically validated measure of the extent of myocardial jeopardy.^6^,^7^

## LGE imaging

LGE plays an important diagnostic and prognostic role in patients with ischaemic heart disease.^8^-^10^ In patients with chronic myocardial infarction scheduled for implantable cardioverterdefibrillator (ICD) implantation, transmural involvement as defined by LGE CMR identifies a subgroup with increased risk for life-threatening arrhythmias and cardiac death.^11^

According to a recent study by Desjardins *et al.*,^12^ ventricular tachycardia (VT) circuits are mainly located in the centre of the LGE CMR-defined infarcts. Total infarct size can be ascertained by LGE CMR and is a strong predictor of future events in patients with coronary artery disease.^13^

The absence of contrast enhancement during the first two minutes after contrast injection in the centre of an area of infarction that may persist on the LGE images points to microvascular obstruction, which is associated with a worse prognosis and outcome.^14^-^16^

## Stress perfusion imaging

Adenosine perfusion CMR has a high diagnostic accuracy in detecting coronary artery stenosis in patients with suspected coronary artery disease (CAD).^17^,^18^ A combined perfusion and infarction CMR examination with a visual interpretation algorithm can accurately diagnose CAD in the clinical setting.^19^ In a recent large, multicentre, multivendor study, the sensitivity of perfusion CMR in detecting CAD was superior to singlephoton emission computed tomography (SPECT), while its specificity was inferior to SPECT.^20^

Adenosine perfusion CMR provides excellent risk stratification and intermediate-term prognostic value in patients with stable CAD.^21^ The presence of a reversible perfusion deficit is associated with a tripled risk for death or non-fatal myocardial infarction.^22^ The presence of abnormal CMR characteristics, including a reversible perfusion deficit, is a strong predictor of myocardial events during follow up.^23^

## Viability assessment

Several different methods of assessing myocardial viability are available in the diagnostic armamentarium. Viability tests have become a crucial tool in evaluating whether patients with congestive cardiac failure related to CAD might benefit from revascularisation therapy.^24^-^26^

Allman *et al.*^27^ demonstrated a strong association between viable myocardium on non-invasive testing and increased survival after revascularisation, with a reduction in annual mortality of 79.6% compared with medical therapy. Three CMR methods exist for the evaluation of viability:

• resting LV wall end-diastolic wall thickness (> 5 mm regarded as viable)^28^
• low-dose dobutamine (LDD) stress assessment of contractile reserve^29^
• LGE of non-viable scar tissue.^30^

According to a recent meta-analysis, LGE CMR provides the highest sensitivity and negative predictive value among the three methods. LDD CMR, however, has the highest specificity and PPV.^31^ If LGE CMR is compared with PET-FDG, rest-distribution thallium-201 SPECT and technetium-99m sestamibi-SPECT, and dobutamine stress echocardiography, it also performs better for predicting functional improvement after revascularisation of hibernating myocardium. LGE CMR also has a higher sensitivity, NPV and PPV than the other available techniques.^25^,^32^

Cardiovascular MRI provides a unique tool to assess multiple interrelated clinical markers of viability in a single test.^29^ The comprehensive assessment of ventricular mass, volume, function and perfusion as well as the ability to establish the presence and extent of non-viable tissue and AAR during a single CMR scan is unparalleled in the diagnostic work-flow of ischaemic heart disease.

## Cardiomyopathies

**Fig. 2.**
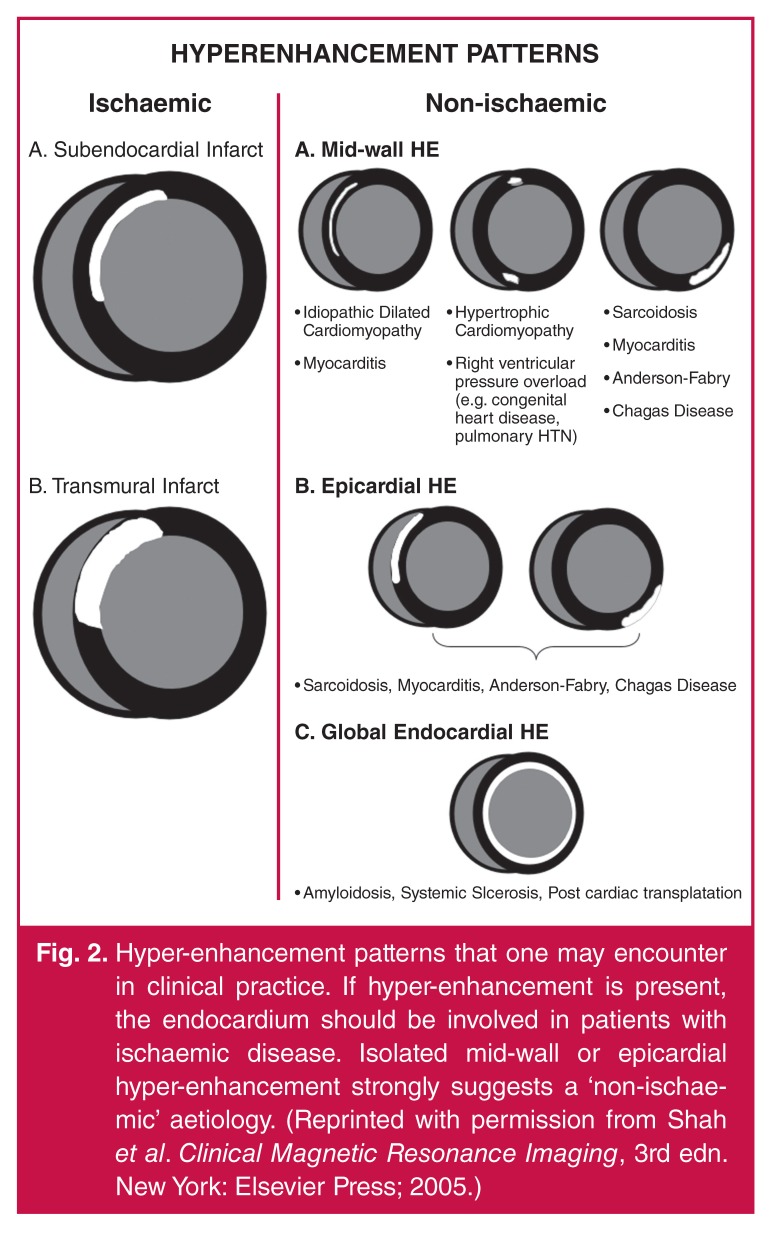
Hyper-enhancement patterns that one may encounter in clinical practice. If hyper-enhancement is present, the endocardium should be involved in patients with ischaemic disease. Isolated mid-wall or epicardial hyper-enhancement strongly suggests a ‘non-ischaemic’ aetiology. (Reprinted with permission from Shah et al. Clinical Magnetic Resonance Imaging, 3rd edn. New York: Elsevier Press; 2005.)

CMR is fast becoming an invaluable tool in the assessment of cardiomyopathies. Regional and global myocardial function can be assessed, and its unique capability to visualise the apex and lateral wall supersedes all other imaging modalities. It is also unique in being able to perform tissue characterisation and to suppress fat, which, combined with LGE, assists in differentiating between various forms of cardiomyopathy (Fig. 2).

Hypertrophic cardiomyopathy (HCM) is the leading cause of sudden death in young people. The majority of HCM patients with sudden cardiac death have few or no clinical symptoms.^33^ CMR is a powerful tool in the diagnosis and risk stratification of HCM; it is widely accepted as the gold standard for assessment of myocardial function as well as left ventricular mass, which has been shown to be a sensitive predictor of adverse outcomes in HCM.^34^

According to a recent meta-analysis, the presence of LGE can predict a higher risk for sudden cardiac death (SCD) and heart failure in patients with HCM, thereby identifying patients who would benefit from ICD placement.^35^ The presence of oedema on T2-weighted CMR has also been observed in patients with HCM.^36^ The presence of LGE has been shown to be a marker for adverse outcomes in several other non-ischaemic cardiomyopathies.^37^,^38^ LGE CMR can help to differentiate between ischaemic and non-ischaemic dilative cardiomyopathy.^39^ According to the revised Task Force criteria for arrhythmogenic right ventricular dysplasia published in *Circulation* in 2010, MRI findings now fall under the major and minor criteria.^40^

Albeit non-specific, CMR findings in ARVD include fatty infiltration of the RV wall, dilatation of the RV, regional or global RV dyskinesis and patchy areas of LGE in the RV wall mainly. CMR shows a characteristic pattern of global sub-endocardiallate enhancement as well as abnormal myocardial and blood-pool kinetics in patients with cardiac amyloidosis.^41^ CMR is a useful diagnostic tool in cardiac involvement owing to sarcoidosis, which is responsible for the majority of deaths resulting from sarcoidosis.^42^,^43^

## Myocarditis

Endomyocardial biopsy (EMB) is considered to be the gold standard for the diagnosis of myocarditis. Recently, CMR has emerged as a promising non-invasive alternative. Three CMR techniques are applied in myocarditis:

• LGE sequences for detection of myocardial necrosis/fibrosis
• T2-weighted images for assessment of myocardial oedema^44^
• T1-weighted sequences before and after contrast injection for the detection of myocardial hyperaemia.

The Lake Louise criteria for CMR diagnosis of myocarditis state that CMR findings are consistent with myocarditis if two out of three of the above criteria are found to be positive.^45^ Although the CMR findings in myocarditis are not specific, they can act as a useful tool for the assessment of myocardial inflammation in patients with suspected acute myocarditis.^46^

## Pericardial disease

CMR is emerging as a most useful tool in the assessment of the pericardium. CMR, owing to its excellent resolution, can comprehensively assess pericardial anatomy. Through evaluation of regional myocardial deformation, ventricular interaction and venous return, CMR can also assess the physiological consequences of pericardial constriction. Owing to its combined anatomical and functional capabilities, CMR is a unique tool that enables one to distinguish between restrictive cardiomyopathy and constrictive pericarditis.

## Congenital heart disease

Echocardiography is the primary diagnostic tool for the assessment of congenital heart disease but CMR can provide valuable information to confirm uncertain diagnoses. The large field of view allows assessment of the anatomical relationships between cardiac and vascular structures.

## Valvular heart disease

Although echocardiography remains the initial tool for assessing cardiac valves, CMR can provide similar information in patients with sub-optimal or unsatisfactory echocardiographic examination.^47^ Valve anatomy and motion as well as the presence of vegetations, thrombi or tumours can be visualised. Velocity measurements can be performed and pressure gradients calculated accurately with good reproducibility. The concomitant excellent determination of ventricular function and volume makes CMR a good alternative when echocardiography is sub-optimal.

## Cardiac masses

The goal of CMR for assessing cardiac and paracardiac masses includes confirming or excluding a mass suspected by X-ray or echocardiography, assessing its location, mobility and relationship to surrounding tissues, and imaging the degree of vascularity; and distinguishing solid lesions from fluid and determining tissue characteristics as well as the specific nature of a mass. Owing to its excellent resolution, tissue characterisation and multiplanar approach, the extent of intra- or pericardial mass lesions can be clearly visualised. The additional administration of gadolinium contrast agents can assess vascularity and help to differentiate tumour from thrombus.

## Coronary artery imaging with CMR

There has been continuous improvement in image quality and examination time in coronary artery imaging with CMR. Hamden *et al.*^48^ recently compared 3.0-T MRI with 64-slice CT angiography of the coronary arteries and concluded that, although both modalities could similarly identify significant coronary stenosis in patients with suspected or known CAD, CT angiography showed a favourable trend towards higher diagnostic performance. CMR is a most useful alternative modality to CT for the detection of anomalous coronary vessels, especially when ionised contrast administration is contra-indicated.^49^

## The future

Imaging speed is likely to continue to increase and larger and larger imaging volumes will become accessible at any given spatial and temporal resolution. The ability to accurately assess total scar burden via T1 mapping could provide a more objective method of non-invasively quantifying diffuse myocardial fibrosis, as recent studies have validated this method in various myocardial diseases.^50^,^51^

Quantitative analysis of perfusion studies will become easier and more applicable in the clinical setting. Myocardial tagging, enabling the CMR specialist to quantify diastolic wall motion will probably move from the research environment into clinical practice. Although coronary artery imaging via CMR is still in its infancy, the imaging of carotid plaque composition looks promising, especially in evaluating the response to lipid-lowering drugs.^52^ Plaque characterisation with 3.0-T MRCA will probably play an important role in the diagnosis and risk stratification of CAD in the future.

## Conclusion

CMR is a unique, versatile, rapidly evolving, non-invasive diagnostic tool offering complete coverage of the heart, and is independent of chest wall anatomy. Owing to its excellent resolution, interstudy reproducibility, user independence and absence of radiation exposure, it is bound to play an increasingly important role in cardiac imaging.

CMR is regarded as the gold standard for evaluating ventricular function, because of its reproducibility and validated assessment of ventricular size, function and mass of both the left and right ventricles. CMR plays an increasingly important role in the work-up of ischaemic heart disease as well as the non-ischaemic cardiomyopathies. Owing to the versatility and multitude of possible sequences, it is necessary to assemble lists of sequences into protocols that are specifically tailored to diagnostic questions or scenarios, in order to decrease scan time, and streamline and simplify the technique.
